# IS SOCIAL ENGAGEMENT RELATED TO FEWER HOSPITALIZATIONS IN COMMUNITY-DWELLING OLDER ADULTS?

**DOI:** 10.1093/geroni/igz038.2769

**Published:** 2019-11-08

**Authors:** Bryan D James, Raj C Shah, Melissa Lamar, Lisa L Barnes, Robert S Wilson, David A Bennett, Julie A Schneider

**Affiliations:** 1 Rush Alzheimer’s Disease Center, Chicago, Illinois, United States; 2 Rush University, Chicago, Illinois, United States

## Abstract

We tested the hypothesis that more socially engaged older adults experience fewer hospitalizations. Data came from 1,153 older adults (72.4% female, mean age 80.8, 14.6 years education at baseline), enrolled in the Rush Memory and Aging Project, with survey data linked to Medicare claims records (mean 5.0 [SD=3.1] years of Medicare coverage after study baseline). Linear regression models were fit with annual rate of hospitalization as outcome with terms for age, sex, and education. Engaging in more social activities (est=-0.16, SE=0.05, p=0.002) and larger life space (est=-0.08, SE=0.03, p=0.005) were associated with a lower rate of hospitalization, while a higher level of loneliness (est=0.18, SE=0.06, p=0.002) was associated with greater rate of hospitalization; size of social networks (est=-0.01, SE=0.01, p=0.069) was not associated with hospitalization. When examined separately by admission type, the same significant associations were found for nonelective (emergency and urgent) hospitalizations, but not for elective hospitalizations. After further adjusting for marital status, baseline levels of depressive symptoms, chronic medical conditions, physical activity, and ADL disabilities, only social activities were significantly related to hospitalization rate (total and nonelective). Adjusting for disability attenuated these associations the most, indicating that functional status may confound the relationship between social engagement and hospitalization. More research is necessary to determine if social engagement in older age can proactively help to keep older adults out of the hospital, or alternatively whether level of social engagement is a marker for functional status or other health factor that is directly related to risk of hospitalization.

